# Super T2-FLAIR mismatch sign: a prognostic imaging biomarker for non-enhancing astrocytoma, IDH-mutant

**DOI:** 10.1007/s11060-024-04758-4

**Published:** 2024-07-12

**Authors:** Iori Ozono, Shumpei Onishi, Ushio Yonezawa, Akira Taguchi, Novita Ikbar Khairunnisa, Vishwa Jeet Amatya, Fumiyuki Yamasaki, Yukio Takeshima, Nobutaka Horie

**Affiliations:** 1 Department of Neurosurgery, Graduate School of Biomedical and Health Sciences, Hiroshima University, 1-2-3 Kasumi, Minami-ku, Hiroshima, 734-8551 Japan; 2 Department of Pathology, Graduate School of Biomedical and Health Sciences, Hiroshima University, Hiroshima, Japan

**Keywords:** Astrocytoma, IDH-mutant, T2-FLAIR mismatch sign, Non-enhancing glioma, Imaging biomarker, Diffuse glioma

## Abstract

**Purpose:**

The T2-FLAIR mismatch sign is a highly specific diagnostic imaging biomarker for astrocytoma, IDH-mutant. However, a definitive prognostic imaging biomarker has yet to be identified. This study investigated imaging prognostic markers, specifically analyzing T2-weighted and FLAIR images of this tumor.

**Methods:**

We retrospectively analyzed 31 cases of non-enhancing astrocytoma, IDH-mutant treated at our institution, and 30 cases from The Cancer Genome Atlas (TCGA)/The Cancer Imaging Archive (TCIA). We defined “super T2-FLAIR mismatch sign” as having a significantly strong low signal comparable to cerebrospinal fluid at non-cystic lesions rather than just a pale FLAIR low-signal tumor lesion as in conventional T2-FLAIR mismatch sign. Cysts were defined as having a round or oval shape and were excluded from the criteria for the super T2-FLAIR mismatch sign. We evaluated the presence or absence of the T2-FLAIR mismatch sign and super T2-FLAIR mismatch sign using preoperative MRI and analyzed the progression-free survival (PFS) and overall survival (OS) by log-rank test.

**Results:**

The T2-FLAIR mismatch sign was present in 17 cases (55%) in our institution and 9 cases (30%) within the TCGA-LGG dataset without any correlation with PFS or OS. However, the super T2-FLAIR mismatch sign was detected in 8 cases (26%) at our institution and 13 cases (43%) in the TCGA-LGG dataset. At our institution, patients displaying the super T2-FLAIR mismatch sign showed significantly extended PFS (122.7 vs. 35.9 months, *p* = 0.0491) and OS (not reached vs. 116.7 months, *p* = 0.0232). Similarly, in the TCGA-LGG dataset, those with the super T2-FLAIR mismatch sign exhibited notably longer OS (not reached vs. 44.0 months, *p* = 0.0177).

**Conclusion:**

The super T2-FLAIR mismatch is a promising prognostic imaging biomarker for non-enhancing astrocytoma, IDH-mutant.

**Supplementary Information:**

The online version contains supplementary material available at 10.1007/s11060-024-04758-4.

## Introduction

The 2016 update of the World Health Organization (WHO) classification of central nervous system (CNS) tumors marked a pivotal shift in brain tumor diagnosis, transitioning from traditional histopathologic classification to molecular genetic classification [1]. This approach decreased the interobserver variation inherent in histological diagnoses [2] and enabled stratification of patient prognosis [3–6]. This approach was further developed in the WHO CNS 5th edition, updated in 2021, and further refined the classification of adult-type diffuse gliomas based on the status of the isocitrate dehydrogenase (IDH) gene and chromosome 1p and 19q [7]. The diffuse gliomas with IDH mutation and 1p/19q non-codeletion are now diagnosed as “astrocytoma, IDH-mutant,” delineating them from “glioblastoma, IDH-wildtype” and “oligodendroglioma, IDH-mutant and 1p/19q codeleted” due to distinct prognostic and clinical/genetic characteristics. The preoperative identification of diffuse gliomas holds substantial clinical value, given that gross total resection significantly enhances prognosis in “astrocytoma, IDH-mutant” compared to other entities [8, 9].

Magnetic resonance imaging (MRI) plays a crucial role in the preoperative diagnosis of tumors and establishing treatment strategies. Recent studies have highlighted the molecular diagnostic significance of MRI findings. Particularly, the T2-weighted Imaging-Fluid-Attenuated Inversion Recovery (T2-FLAIR) mismatch sign has been established as a diagnostic imaging biomarker for astrocytoma, IDH-mutant with a high predictive value and specificity [10–12]. However, previous studies found no correlation between the T2-FLAIR mismatch sign and progression-free survival (PFS) or overall survival (OS) [10, 13]. Several clinical, molecular, and pathological prognostic factors for patients with IDH-mutant astrocytoma have been identified, including age, tumor size, primary tumor site, surgical outcome, mitotic index, and CDKN2A/B homozygous deletion [14–16]. The CDKN2A/B homozygous deletion has been incorporated into the grading criteria for these tumors, leading to the classification of tumors with CDKN2A/B deletion as WHO grade 4 [7]. Various imaging biomarkers have been explored to differentiate WHO grade 4 from WHO grade 2–3 astrocytoma with IDH mutation. These include gadolinium enhancement, low apparent diffusion coefficient, high cerebral tumor blood volume, and intratumoral susceptibility signal intensity observed in gadolinium-enhanced T1-weighted imaging, diffusion-weighted imaging, perfusion-weighted imaging, and susceptibility-weighted imaging, respectively [17]. However, prognostic imaging biomarkers for predicting WHO grade 2–3 astrocytoma, specifically non-enhancing astrocytoma, IDH-mutant are limited.

This study aimed to develop a useful prognostic imaging biomarker of non-enhancing astrocytoma, IDH-mutant. Our review encompassed our institutional case series and data from The Cancer Genome Atlas (TCGA)/The Cancer Imaging Archive (TCIA) pertaining to astrocytoma, IDH-mutant. We focused on the observation of a conspicuously low-intensity lesion in FLAIR imaging, occasionally encountered in this tumor, and named “super T2-FLAIR mismatch sign” and investigated the prognostic value of this sign.

## Materials and methods

This retrospective study was approved by our institutional review board (E2022-0038). This study utilized data from two cohorts; the patients treated at our institute and publicly available data from TCGA/TCIA, TCGA-lower grade glioma (LGG) [18]. The patients who fulfilled the following criteria were included in this study: (i) newly diagnosed astrocytoma, IDH-mutant; (ii) tumors with WHO grade 2 or 3. (iii) Participants with available preoperative MR images with FLAIR, T2WI, T1-weighted imaging (T1WI) and post-contrast T1WI. (iv) Tumors with no evident gadolinium contrast enhancement on post-contrast T1WI.

### Patient data and molecular pathological analysis in our institution

Between October 2009 and September 2022, 31 patients met the eligibility criteria. Pertinent clinical data, including age at diagnosis, gender, Karnofsky performance status (KPS), tumor WHO grade, extent of tumor resection (total or non-total), administration of radiation therapy, chemotherapy using temozolomide, PFS, and OS, were collected from medical records.

Surgically resected tumor specimens underwent fixation in 10% phosphate-buffered formalin and subsequent embedding in paraffin blocks. Standard histological diagnosis was conducted by staining representative slides with hematoxylin-eosin reagent. Immunohistochemical staining for IDH1-R132H and α-thalassemia X-linked intellectual disability (ATRX) was performed using an automated immunostainer (BenchMark GX; Ventana). 1p/19q co-deletions were detected through fluorescence in situ hybridization analysis on paraffin-embedded tumor tissue samples [19]. In our study, direct confirmation of CDKN2A/B homozygous deletion has not been obtained. However, we have assessed the S-methyl-50-thioadenosine phosphorylase (MTAP) immunostaining, which has been reported as an excellent surrogate marker. Cases with negative MTAP staining were excluded as they were diagnosed as astrocytoma, grade 4, according to WHO 2021 criteria [20]. Tumors were diagnosed based on WHO CNS 5th edition [7].

### MR acquisition and evaluation

MR scans were acquired using 3.0 T scanners (Ingenia CX 3.0 T; Philips Healthcare, Best, Netherlands, or Signa Excite HD 3.0 T; GE Medical Systems, Milwaukee, WI, USA). Preoperative T1WI, T2WI, FLAIR, and post-contrast T1WI sequencing were each evaluated by two board-certified neurosurgeons (I.O. and S.O.). The imaging parameters are described in Supplementary Material 1.

### Patient data in TCGA-LGG

MRI was available for 199 cases of LGGs in the TCIA database. Clinical information (age, gender, PFS, OS), histopathology, WHO grade, and molecular classifications were obtained from the Supplementary Material of a previously published article [21]. This study included 30 cases of LGG with IDH-mutant and 1p/19q non-codeleted, which can be considered astrocytoma, IDH-mutant. The other molecular types of LGG (101 cases), cases without appropriate images (37 cases), and cases with obvious contrast enhancement (31 cases) were excluded.

### Definition of conventional T2-FLAIR mismatch sign and “super T2-FLAIR mismatch sign”

The conventional T2-FLAIR mismatch sign was designated as per the original definition [10]; the presence of a complete/near-complete hyperintense signal on T2WI and a relatively hypointense signal on FLAIR except for a hyperintense peripheral rim. Based on a previous article, a positive T2-FLAIR mismatch sign was defined as a tumor with > 50% T2-FLAIR mismatch lesion [22].

We defined the ‘super T2-FLAIR mismatch sign’ as a tumor displaying a complete or near-complete hyperintense signal on T2-weighted imaging (T2WI) while exhibiting significantly strong low intensity comparable to cerebrospinal fluid. This characteristic contrasts with the conventional T2-FLAIR mismatch, which typically presents as a pale FLAIR low-intensity tumor lesion. Additionally, cysts possessing well-defined margins of a round or oval shape within the tumor were excluded from the super T2-FLAIR mismatch sign criteria. In our study, the super T2-FLAIR mismatch sign was identified by the presence of the lesion with a diameter measuring 5 mm or larger.

The two reviewers (I.O. with 9 years of experience and S.O. with 11 years of experience) were blinded to each other’s results. The inter-reviewer agreement was evaluated using the Kappa statistic (κ = 0–0.40, poor; κ = 0.41–0.60, moderate; κ = 0.61–0.80, good; κ = 0.81–1.00, excellent). In the event of disagreement, two reviewers discussed the findings, and a final consensus was reached with the involvement of a senior neurosurgeon (F.Y. with 30 years of experience).

### Statistical analysis

PFS was defined as the time from initial surgery to the tumor progression or tumor-related death. OS was defined as the time from initial surgery to the tumor-related death. The PFS and OS of the patients were analyzed using a Kaplan–Meier analysis. The patients were divided into two groups based on the presence or absence of the conventional T2-FLAIR mismatch sign and the super T2-FLAIR mismatch sign, and the PFS and OS were compared using log-rank tests. The patients’ characteristics and clinical information were compared using Fisher’s exact test. All statistical analyses were performed using JMP^®^ Pro version 17 (SAS Institute, Cary, NC, USA). P-values of less than 0.05 indicated statistical significance.

## Results

### Clinical and radiological characteristics of our institution dataset

The mean age of the 31 cases was 40.6 ± 12.7 years, comprising 10 females and 21 males. Among the included patients, 25 (81%) cases were classified as WHO grade 2, while 6 (19%) were designated as WHO grade 3. In terms of treatment, total resection was accomplished in 10 (32%) cases, 21 (68%) cases received radiation therapy, and temozolomide was administered in 19 (61%) cases.

**Fig. 1:**
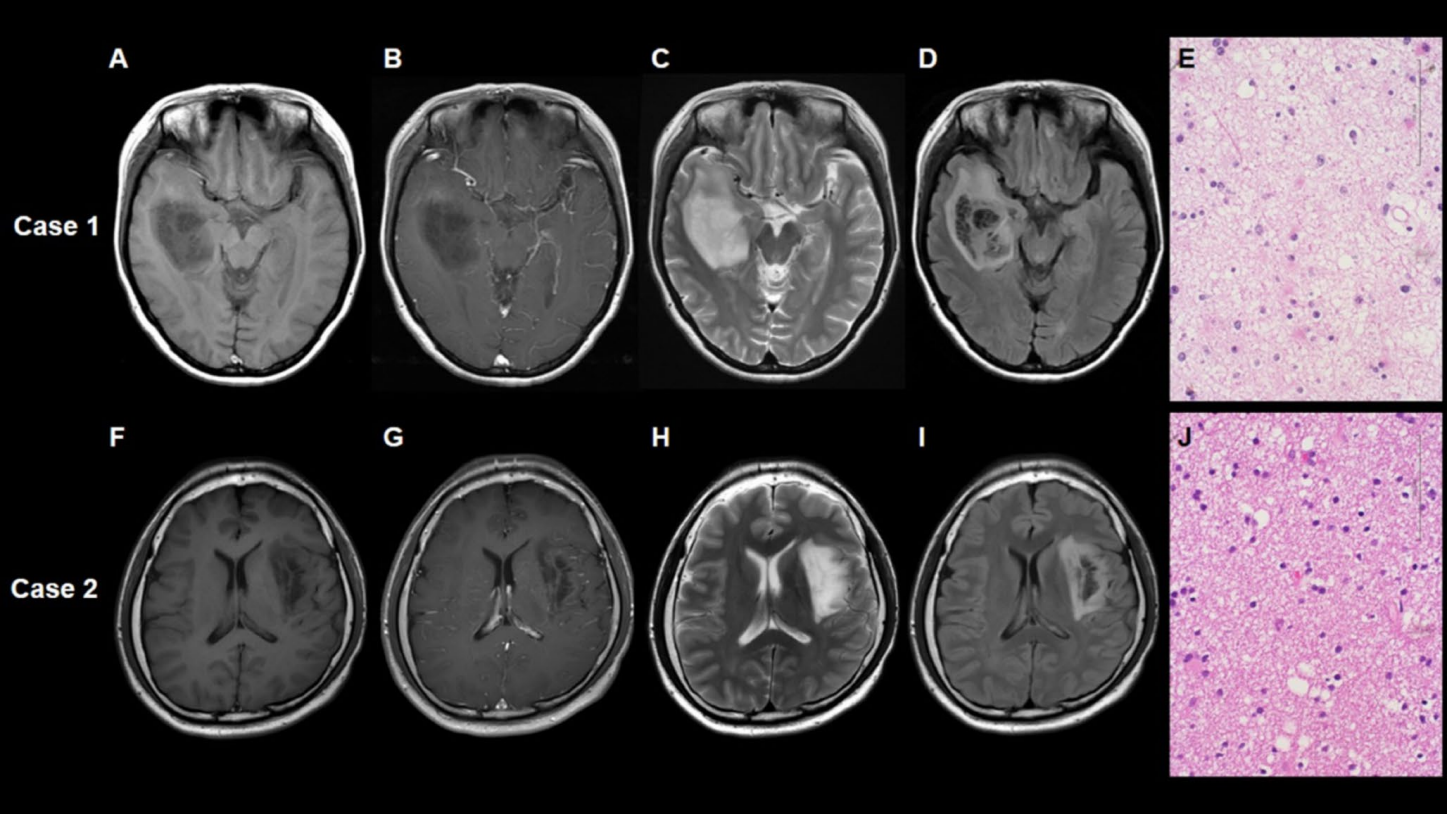
Case 1 is a representative case with right frontal lobe astrocytoma, IDH-mutant featuring both the positive super T2-FLAIR mismatch sign and positive conventional T2-FLAIR mismatch sign. No evident contrast enhancement was observed (**A**, **B**). The T2-weighted image **(C**) illustrates a tumor exhibiting nearly uniform hyperintensity. Notably, the FLAIR image (**D**) reveals a distinctive area within the tumor displaying significantly strong low intensity, comparable to cerebrospinal fluid. Hematoxylin-eosin staining (**E**) showed enlarged intercellular space and a prominent microcystic background. Case 2 is a representative case with left insular astrocytoma, IDH-mutant displaying a positive super T2-FLAIR mismatch sign but a negative conventional T2-FLAIR mismatch sign. No evident contrast enhancement was observed (**F**, **G**). The T2-weighted image **(H**) illustrates a tumor exhibiting nearly uniform hyperintensity. In the FLAIR image (**I**), a distinct area within the tumor shows significantly strong low intensity, akin to cerebrospinal fluid. The size of the area with high-intensity T2WI but low-intensity FLAIR did not exceed 50% of the entire tumor. Hematoxylin-eosin staining (**J**) shows enlarged intercellular space and a prominent microcystic background.

**Fig. 2:**
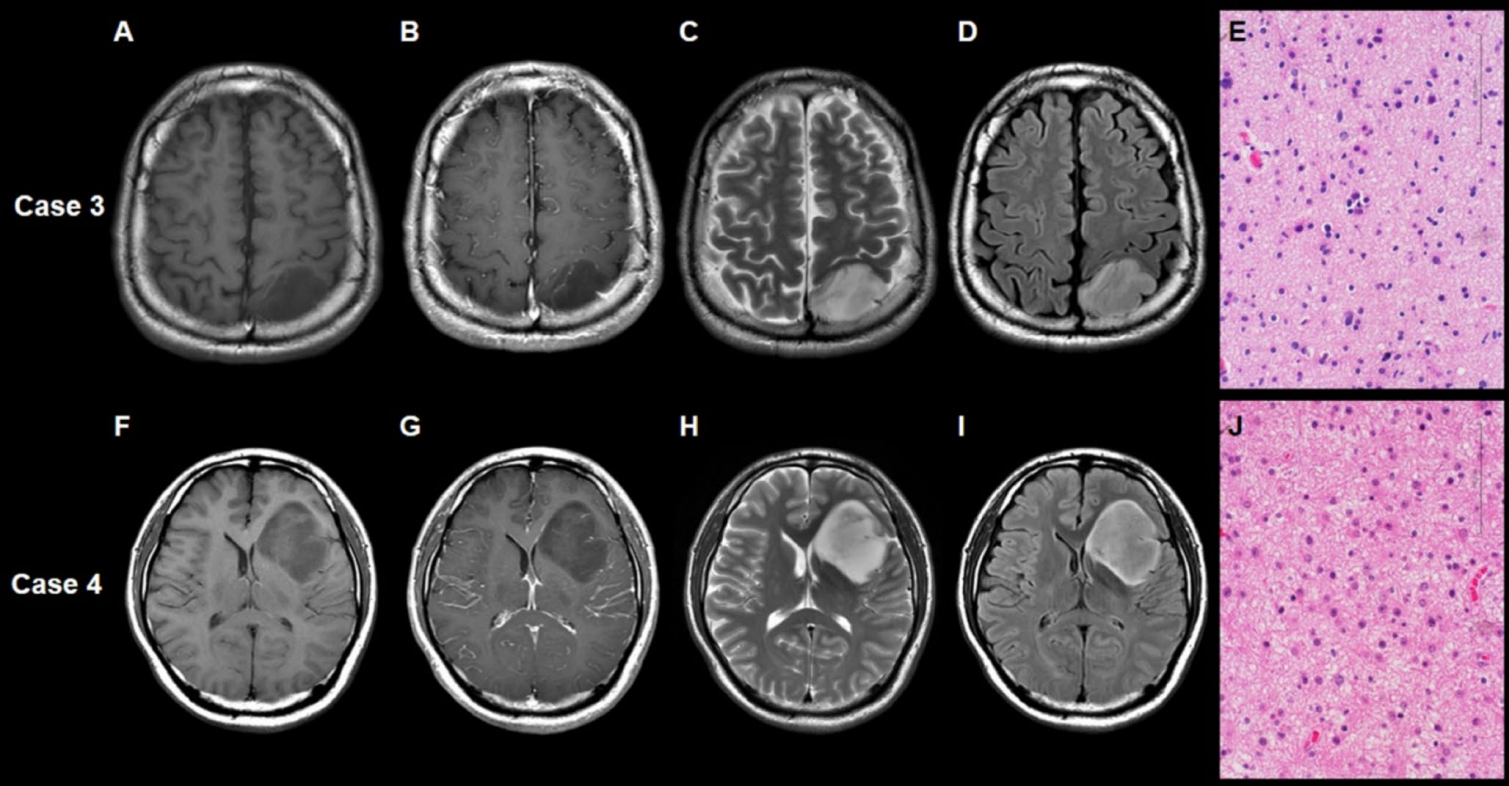
Case 3 is a representative case with left occipital lobe astrocytoma, IDH-mutant displaying the negative super T2-FLAIR mismatch sign and negative conventional T2-FLAIR mismatch sign. No evident contrast enhancement is observed (**A**, **B**). The T2-weighted (**C**) and FLAIR (**D**) images illustrate a tumor displaying a uniform hyperintensity. Hematoxylin-eosin staining showed (**E**) pleomorphic astrocyte tumor cells with a microcystic background. Case 4 is the representative case with left frontal lobe astrocytoma, IDH-mutant displaying a negative super T2-FLAIR mismatch sign but a positive conventional T2-FLAIR mismatch sign. No observable contrast enhancement was noted (**F**, **G**). The tumor manifests as a hyperintense area in the T2-weighted image (**H**) and exhibits a relatively low signal on the FLAIR image (**I**), except for a hyperintense peripheral rim. Hematoxylin-eosin staining (**J**) shows an astrocytic tumor amidst a fibrillary and microcystic background.

The T2-FLAIR mismatch sign and super T2-FLAIR mismatch sign were presented in 17 cases (55%) and 8 cases (26%), respectively. Inter-reviewer agreement for these mismatch signs was excellent (κ = 1.000, 1.000, respectively). Supplementary Material 2 shows the characteristics of our dataset according to the status of the T2-FLAIR mismatch sign. The T2-FLAIR mismatch sign-positive cases were more frequent at WHO grade 2 in our dataset (*p* = 0.0671). Other factors, including age, gender, and extent of resection, were not associated with this sign. Table 1 shows the characteristics of our dataset according to the status of the super T2-FLAIR mismatch sign. No significant difference in WHO grade was observed between positive and negative super T2-FLAIR mismatch signs (*p* = 0.2976). The representative case with the positive super T2-FLAIR mismatch sign and positive conventional T2-FLAIR mismatch sign is shown as Case 1 (Fig. 1). The representative case with the positive super T2-FLAIR mismatch sign but negative conventional T2-FLAIR mismatch sign is shown as Case 2 (Fig. 1). The representative case of the negative super T2-FLAIR mismatch sign and negative conventional T2-FLAIR mismatch sign is shown as Case 3 (Fig. 2). The representative case of the negative super T2-FLAIR mismatch sign but positive conventional T2-FLAIR mismatch sign is shown as Case 4 (Fig. 2).

**Table 1:** Characteristics of the patients with astrocytoma, IDH-mutant according to the super T2-FLAIR mismatch sign

	Our dataset (*n* = 31)					TCGA (*n* = 30)				
	Super T2-FLAIR mismatch sign									
	Positive (*n* = 8)		Negative (*n* = 23)		*p*-value	Positive (*n* = 13)		Negative (*n* = 17)		*p*-value
Age at diagnosis (years)	40.2 ± 13.5		41.8 ± 9.9		0.7644	39.7 ± 11.6		37.2 ± 13.7		0.5982
Gender					0.3809					0.0634
Female	4	(50%)	6	(26%)		9	(69%)	5	(29%)	
Male	4	(50%)	17	(74%)		4	(31%)	12	(71%)	
KPS (%)					1.0000					
< 90	1	(13%)	5	(22%)						
* ≥* 90	7	(88%)	18	(78%)						
WHO grade					0.2976					0.0191*
2	8	(100%)	17	(74%)		11	(85%)	8	(47%)	
3	0	(0%)	6	(26%)		1	(8%)	9	(53%)	
Extent of resection					0.2216					
Total	1	(13%)	9	(39%)						
Non-total	7	(88%)	14	(61%)						
Radiation therapy	7	(88%)	14	(61%)	0.2216	9	(69%)	15	(88%)	0.3598
Temozolomide	7	(88%)	12	(52%)	0.1082					

FLAIR, fluid-attenuated inversion recovery; IDH, isocitrate dehydrogenase; KPS, Karnofsky performance status; TCGA, The Cancer Genome Atlas; WHO, World Health Organization

* *p* < 0.05

### Prognostic analysis of mismatch signs in our dataset

**Fig. 3:**
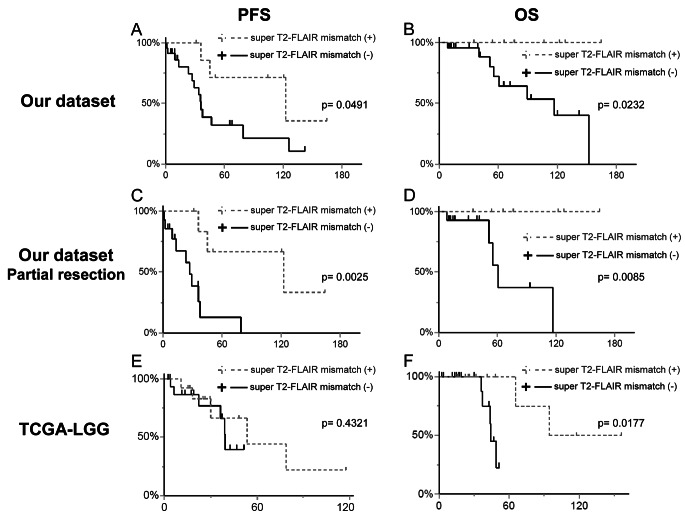
Kaplan–Meier analyses of the median progression-free survival (PFS) and overall survival (OS). In our dataset, the cases with the super T2-FLAIR mismatch sign showed significantly longer PFS (122.7 months vs. 35.9 months, *p* = 0.0491) and longer OS (not reached vs. 116.7 months, *p* = 0.0232) (**A**, **B**). Among the patients with non-total resection, the super T2-FLAIR mismatch sign was also associated with significantly longer PFS (122.7 vs. 27.0 months, *p* = 0.0025) and OS (not reached vs. 60.7 months, *p* = 0.0085) (**C**, **D**). In the TCGA-LGG dataset, the cases with the super T2-FLAIR mismatch sign did not show a significant correlation with PFS (53.6 months vs. 39.4 months, *p* = 0.4321) but were significantly associated with a longer OS (not reached vs. 44.0 months, *p* = 0.0177) (**E**, **F**).

We further assessed the prognostic significance of both the T2-FLAIR mismatch sign and the super T2-FLAIR mismatch sign. The presence of the T2-FLAIR mismatch sign did not demonstrate a significant association with either PFS (79.1 months vs. 33.6 months, *p* = 0.1624) or OS (not reached vs. 152 months, *p* = 0.3153). Conversely, cases featuring a positive super T2-FLAIR mismatch sign exhibited significantly extended PFS (122.7 months vs. 35.9 months, *p* = 0.0491) and OS (not reached vs. 116.7 months, *p* = 0.0232, Fig. 3A, B). Further analysis focusing on patients with residual tumor post-surgery revealed that among those with non-total resection, a positive super T2-FLAIR mismatch sign was significantly associated with prolonged PFS (122.7 vs. 27.0 months, *p* = 0.0025) and OS (not reached vs. 60.7 months, *p* = 0.0085, Fig. 3C, D).

### Clinical characteristics of TCGA-LGG

We investigated the patients in the TCGA-LGG dataset. The mean age of the 30 cases was 38.3 ± 12.9 years, consisting of 15 females and 16 males. Among the included patients, 19 (63%) cases were WHO grade 2, 10 (33%) cases were WHO grade 3, and 1 (3%) case was unknown. Twenty-four (80%) cases underwent radiation therapy.

The positive T2-FLAIR and super T2-FLAIR mismatch signs were observed in 9 (30%) and 13 cases (43%), respectively. Inter-reviewer agreement for these mismatch signs was excellent (κ = 1.000, 1.000, respectively). Supplementary Material 2 shows the characteristics of the TCGA-LGG dataset according to the status of the T2-FLAIR mismatch sign. The factors including age, gender, WHO grade, and receiving radiation therapy were not associated with this sign. The characteristics of patients regarding the status of the super T2-FLAIR mismatch sign at the TCGA-LGG dataset were also summarized in Table 1. A significant difference was observed in WHO grade: 11 (85%) cases were WHO grade 2 and 1 (8%) case was WHO grade 3 in the positive group, and 8 (44%) cases were WHO grade 2 and 10 (56%) cases were WHO grade 3 in the negative group, respectively (*p* = 0.0191, Fisher’s exact test). We further combined our dataset and TCGA-LGG and observed that the tumors with positive super T2-FLAIR mismatch sign were significantly more likely to be WHO grade 2 (*p* = 0.0114, Fisher’s exact test).

### Prognostic analysis of mismatch signs in TCGA-LGG

Finally, we analyzed the prognostic value of the T2-FLAIR mismatch sign and super T2-FLAIR mismatch sign using TCGA-LGG dataset. The T2-FLAIR mismatch sign was not associated with both PFS (53.6 months vs. 39.4 months, *p* = 0.2558) and OS (94.5 vs. 49.0 months, *p* = 0.5587). While the cases with the super T2-FLAIR mismatch sign did not show a significant association with PFS (53.6 months vs. 39.4 months, *p* = 0.4321), they were significantly associated with a longer OS (not reached vs. 44.0 months, *p* = 0.0177; Fig. 3E, F). We further combined our dataset and TCGA-LGG dataset and analyzed the overall survival. The positive super T2-FLAIR mismatch sign was a favorable prognostic marker and was significantly associated with a longer OS (not reached vs. 60.7 months, *p* = 0.0050; Supplementary Material 3).

## Discussion

The T2-FLAIR mismatch sign has been widely accepted as the specific imaging biomarker for the diagnosis of astrocytoma, IDH-mutant among adult-type diffuse lower-grade glioma. Our study revealed that the positive “super T2-FLAIR mismatch sign” was a better prognostic marker in patients with non-enhancing astrocytoma, IDH-mutant WHO grade 2 and 3.

Although the definition of the T2-FLAIR mismatch sign involves a hyperintense signal on T2WI and a relatively hypointense signal on FLAIR, the specific signal intensity on FLAIR remains undefined and can vary among cases classified as T2-FLAIR mismatch sign positive. This study was focused on the signal intensity observed in FLAIR imaging due to the lack of a definitive standard for FLAIR signal intensity in identifying the T2-FLAIR mismatch sign. We introduced the concept of the ‘super T2-FLAIR mismatch sign,’ characterized by a notably strong low intensity comparable to cerebrospinal fluid on FLAIR.

The T2-FLAIR mismatch sign was exhibited not only in astrocytoma, IDH-mutant, but also in pilomyxoid astrocytoma, H3K27M mutant midline glioma, and dysembryoplastic neuroepithelial tumor (DNET) [23, 24]. Before the formal designation of the T2-FLAIR mismatch sign, it was previously recognized as the ‘FLAIR ring sign’ within DNET [25]. DNET could contain a region displaying significantly strong low intensity similar to cerebrospinal fluid on FLAIR images. These portions are known as pseudo-cysts [26] because they comprise oligodendroglioma-like cells within a mucin-rich background alongside glioneuronal elements featuring floating neurons [27]. The pathological analysis of conventional T2-FLAIR mismatch sign showed characteristic microscopical microcystic changes at the T2-FLAIR mismatch lesion [10, 13, 28], while the T2-FLAIR matched lesion showed a tumor with high cellularity [13, 28]. Yamashita et al. categorized astrocytoma, IDH-mutant into three groups based on the presence or absence of the T2-FLAIR mismatch sign and pathological findings of microcysts. They demonstrated a significant association between the expansion of intercellular spaces, which included microcysts, and the T2-FLAIR mismatch [29]. Moreover, they also suggested the possibility that increased fluid in the enlarged intercellular spaces could result in more effective suppression on FLAIR images. These findings suggest that the suppression signal observed on the FLAIR image could be attributed to the low cellularity and the expanded intercellular space within the tumor. The presence of the ‘super T2-FLAIR mismatch sign,’ characterized by FLAIR suppression akin to cerebrospinal fluid, might denote an even more pronounced reduction in cellularity compared to the standard T2-FLAIR mismatch sign, as demonstrated in our representative cases.

Although IDH-mutant astrocytoma with T2-FLAIR mismatch sign showed longer survival, it was not statistically significant [13, 29]. Our results using our institutional dataset and TCGA-LGG dataset were consistent with previous reports. Super T2-FLAIR mismatch sign showed a significantly good prognosis in our and TCGA-LGG datasets. This favorable prognosis might be attributed to lower cellularity, suggesting reduced mitotic activity and lower malignancy. The extent of resection is one of the important prognostic factors of astrocytoma, IDH-mutant [8]. Hence, we further analyzed the prognostic value among patients unable to achieve total tumor resection, confirming a significant association between a positive super T2-FLAIR mismatch sign and a favorable prognosis.

In this study, we validated the prognostic value of the super T2-FLAIR mismatch sign using the TCGA-LGG dataset. Consistent with our findings, patients exhibiting the super T2-FLAIR mismatch sign demonstrated longer OS within the TCGA-LGG dataset. However, no significant difference in PFS was observed based on the status of the super T2-FLAIR mismatch sign in this dataset. Detailed treatment information was unavailable from the TCGA-LGG dataset, warranting further studies to ascertain and confirm the practical utility of the super T2-FLAIR mismatch sign.

A limitation of this study was the small sample size of both our own institution and the TCGA/TCIA datasets, rendering multivariate analysis impossible. Comparison among the four groups could not be evaluated due to the small sample size. This comparison involved dividing patients into those who tested positive and negative for both conventional and super T2-FLAIR mismatch. Our institutional cases did not include minor mutations of IDH, such as IDH1-R132S, IDH1-R132S, and IDH2-R172K. The TCGA dataset also did not provide information on the specific IDH mutation status. Therefore, we were unable to assess the imaging differences between the IDH1-R132H mutation and other minor IDH mutations. The TCGA LGG dataset did not include information about CDKN2A/B and might be at risk of contamination regarding the presence of astrocytoma, WHO grade 4 cases based on WHO 2021 criteria. Our institutional dataset carried the same risk because we did not directly confirm CDKN2A/B homozygous deletion and instead used a surrogate marker such as MTAP immunostaining. Furthermore, due to the lack of accurate sampling of the super T2-FLAIR mismatch areas, the accurate correlation between the ‘super T2-FLAIR mismatch sign’ and pathological assessment could not be established in this study. The molecular distinction between negative and positive super T2-FLAIR mismatch signs remains unestablished. Larger-scale studies are required to validate and ascertain the efficacy of the super T2-FLAIR mismatch sign.

## Conclusion

The presence of a super T2-FLAIR mismatch sign demonstrated a significant association with improved prognosis in patients diagnosed with non-enhancing astrocytoma, IDH-mutant. This finding underscores the potential of the super T2-FLAIR mismatch sign as a promising prognostic imaging biomarker for non-enhancing astrocytoma, IDH-mutant.

### Electronic supplementary material

Below is the link to the electronic supplementary material.

Supplementary Material 1

Supplementary Material 2

Supplementary Material 3

## Data Availability

No datasets were generated or analysed during the current study.

## References

[1] Louis DN, Perry A, Reifenberger G et al (2016) The 2016 World Health Organization classification of tumors of the central nervous system: a summary. Acta Neuropathol 131:803–820. 10.1007/s00401-016-1545-1 27157931 10.1007/s00401-016-1545-1

[2] Van den Bent MJ (2010) Interobserver variation of the histopathological diagnosis in clinical trials on glioma: a clinician’s perspective. Acta Neuropathol 120:297–304. 10.1007/s00401-010-0725-7 20644945 10.1007/s00401-010-0725-7 PMC2910894

[3] The Cancer Genome Atlas Research Network (2015) Comprehensive, integrative genomic analysis of diffuse lower-grade gliomas. N Engl J Med 372:2481–2498. 10.1056/NEJMoa1402121 26061751 10.1056/NEJMoa1402121 PMC4530011

[4] Dubbink HJ, Atmodimedjo PN, Kros JM et al (2016) Molecular classification of anaplastic oligodendroglioma using next-generation sequencing: a report of the prospective randomized EORTC brain Tumor Group 26951 phase III trial. Neuro Oncol 18:388–400. 10.1093/neuonc/nov182 26354927 10.1093/neuonc/nov182 PMC4767239

[5] Labussière M, Di Stefano AL, Gleize V et al (2014) Tert promoter mutations in gliomas, genetic associations and clinico-pathological correlations. Br J Cancer 111:2024–2032. 10.1038/bjc.2014.538 25314060 10.1038/bjc.2014.538 PMC4229642

[6] Reuss DE, Sahm F, Schrimpf D et al (2015) ATRX and IDH1-R132H immunohistochemistry with subsequent copy number analysis and IDH sequencing as a basis for an integrated diagnostic approach for adult astrocytoma, oligodendroglioma and glioblastoma. Acta Neuropathol 129:133–146. 10.1007/s00401-014-1370-3 25427834 10.1007/s00401-014-1370-3

[7] Louis DN, Perry A, Wesseling P et al (2021) The 2021 WHO classification of tumors of the central nervous system: a summary. Neurooncology 23:1231–1251. 10.1093/neuonc/noab106 10.1093/neuonc/noab106 PMC8328013 34185076

[8] Kawaguchi T, Sonoda Y, Shibahara I et al (2016) Impact of gross total resection in patients with WHO grade III glioma harboring the IDH 1/2 mutation without the 1p/19q co-deletion. J Neurooncol 129:505–514. 10.1007/s11060-016-2201-2 27401154 10.1007/s11060-016-2201-2

[9] Wahner HCW, Träger M, Bender K et al (2020) Predicting survival in anaplastic astrocytoma patients in a single-center cohort of 108 patients. Radiat Oncol 15:282. 10.1186/s13014-020-01728-8 33334378 10.1186/s13014-020-01728-8 PMC7745461

[10] Patel SH, Poisson LM, Brat DJ et al (2017) T2-FLAIR mismatch, an imaging biomarker for IDH and 1p/19q status in Lower-grade gliomas: a TCGA/TCIA project. Clin Cancer Res 23:6078–6085. 10.1158/1078-0432.CCR-17-0560 28751449 10.1158/1078-0432.CCR-17-0560

[11] Jain R, Johnson DR, Patel SH et al (2020) Real world’ use of a highly reliable imaging sign: T2-FLAIR mismatch for identification of IDH mutant astrocytomas. Neuro Oncol 22:936–943. 10.1093/neuonc/noaa041 32064507 10.1093/neuonc/noaa041 PMC7339896

[12] Broen MPG, Smits M, Wijnenga MMJ et al (2018) The T2-FLAIR mismatch sign as an imaging marker for non-enhancing IDH-mutant, 1p/19q-intact lower-grade glioma: a validation study. Neuro Oncol 20:1393–1399. 10.1093/neuonc/noy048 29590424 10.1093/neuonc/noy048 PMC6120363

[13] Deguchi S, Oishi T, Mitsuya K et al (2020) Clinicopathological analysis of T2-FLAIR mismatch sign in lower-grade gliomas. Sci Rep 10:10113. 10.1038/s41598-020-67244-7 32572107 10.1038/s41598-020-67244-7 PMC7308392

[14] Appay R, Dehais C, Maurage CA et al (2019) CDKN2A homozygous deletion is a strong adverse prognosis factor in diffuse malignant IDH-mutant gliomas. Neuro Oncol 21:1519–1528. 10.1093/neuonc/noz124 31832685 10.1093/neuonc/noz124 PMC7145561

[15] Kros JM, Rushing E, Uwimana AL et al (2023) Mitotic count is prognostic in IDH mutant astrocytoma without homozygous deletion of CDKN2A/B. results of consensus panel review of EORTC trial 26053 (CATNON) and EORTC trial 22033–26033. Neurooncology 25:1443–1449. 10.1093/neuonc/noac282 10.1093/neuonc/noac282 PMC10398806 36571817

[16] Liu S, Liu X, Zhuang W (2021) Prognostic factors associated with survival in patients with diffuse astrocytoma. Front Surg 8:712350. 10.3389/fsurg.2021.712350 34722621 10.3389/fsurg.2021.712350 PMC8554054

[17] Yang X, Xing Z, She D et al (2022) Grading of IDH-mutant astrocytoma using diffusion, susceptibility and perfusion-weighted imaging. BMC Med Imaging 22:105. 10.1186/s12880-022-00832-3 35644621 10.1186/s12880-022-00832-3 PMC9150301

[18] The cancer imaging archive (2023) https://public.cancerimagingarchive.net/ncia/login.jsf. Accessed

[19] Woehrer A, Sander P, Haberler C et al (2011) FISH-based detection of 1p 19q co-deletion in oligodendroglial tumors: procedures and protocols for neuropathological practice - a publication under the auspices of the Research Committee of the European Confederation of Neuropathological Societies (Euro-CNS). Clin Neuropathol 30:47–55. 10.5414/npp30047 21329613 10.5414/npp30047

[20] Maragkou T, Reinhard S, Jungo P et al (2023) Evaluation of MTAP and p16 immunohistochemical deficiency as surrogate marker for CDKN2A/B homozygous deletion in gliomas. Pathology 55:466–477. 10.1016/j.pathol.2023.01.005 37032198 10.1016/j.pathol.2023.01.005

[21] Ceccarelli M, Barthel FP, Malta TM et al (2016) Molecular profiling reveals biologically discrete subsets and pathways of progression in diffuse glioma. Cell 164:550–563. 10.1016/j.cell.2015.12.028 26824661 10.1016/j.cell.2015.12.028 PMC4754110

[22] Lasocki A, Gaillard F, Gorelik A, Gonzales M (2018) MRI features can predict 1p/19q status in intracranial gliomas. AJNR Am J Neuroradiol 39:687–692. 10.3174/ajnr.A5572 29519793 10.3174/ajnr.A5572 PMC7410766

[23] Onishi S, Amatya VJ, Kolakshyapati M et al (2020) T2-FLAIR mismatch sign in dysembryoplasticneuroepithelial tumor. Eur J Radiol 126:108924. 10.1016/j.ejrad.2020.108924 32193035 10.1016/j.ejrad.2020.108924

[24] Johnson DR, Kaufmann TJ, Patel SH et al (2019) There is an exception to every rule-T2-FLAIR mismatch sign in gliomas. Neuroradiology 61:225–227. 10.1007/s00234-018-2148-4 30565056 10.1007/s00234-018-2148-4

[25] Parmar HA, Hawkins C, Ozelame R et al (2007) Fluid-attenuated inversion recovery ring sign as a marker of dysembryoplastic neuroepithelial tumors. J Comput Assist Tomogr 31:348–353. 10.1097/01.rct.0000243453.33610.9d 17538277 10.1097/01.rct.0000243453.33610.9d

[26] Rahim S, Ud Din N, Abdul-Ghafar J et al (2023) Clinicopathological features of dysembryoplastic neuroepithelial tumor: a case series. J Med Case Rep 17:327. 10.1186/s13256-023-04062-1 37525202 10.1186/s13256-023-04062-1 PMC10391907

[27] Suh YL (2015) Dysembryoplastic neuroepithelial tumors. J Pathol Transl Med 49:438–449. 10.4132/jptm.2015.10.05 26493957 10.4132/jptm.2015.10.05 PMC4696533

[28] Fujita Y, Nagashima H, Tanaka K et al (2021) The histopathologic and radiologic features of T2-FLAIR mismatch sign in IDH-Mutant 1p/19q non-codeleted astrocytomas. World Neurosurg 149:e253–e260. 10.1016/j.wneu.2021.02.042 33610870 10.1016/j.wneu.2021.02.042

[29] Yamashita S, Takeshima H, Kadota Y et al (2022) T2-fluid-attenuated inversion recovery mismatch sign in lower grade gliomas: correlation with pathological and molecular findings. Brain Tumor Pathol 39:88–98. 10.1007/s10014-022-00433-6 35482260 10.1007/s10014-022-00433-6

