# Supplementary material for: Super T2-FLAIR mismatch sign: a prognostic imaging biomarker for non-enhancing astrocytoma, IDH-mutant
Source: J Neurooncol. 2024 Jul 12;169(3):571–9. doi: 10.1007/s11060-024-04758-4 (PMC11341624; doi:10.1007/s11060-024-04758-4)
Supplement: Supplementary file 1 — Supplementary Material 1 [file 11060_2024_4758_MOESM1_ESM.docx]

**Supplementary Material 1:** Details of MRI parameters

Details of MRI parameters for Ingenia CX 3.0 T, Philips Healthcare

|  | T1WI | T2WI | FLAIR |
| --- | --- | --- | --- |
| repetition time [TR] | 500 ms | 3,000 ms | 10,000 ms |
| echo time [TE] | 10 ms | 100 ms | 130 ms |
| flip angle [FA] | (-) | (-) | (-) |
| echo train length | (-) | 15 | (-) |
| inversion recovery time [TI] | (-) | (-) | 2600 ms |
| field of view [FOV] | 220 mm,  RFOV: 82.03% | 220 mm,  RFOV 80% | 220 mm,  RFOV 78.91% |
| matrix scan | 288 | 512 | 288 |
| reconstruction | 512 | 600 | 512 |
| number of excitations [NEX] | 1 | 2 | 1 |
| section thickness | 5 mm | 5 mm | 5 mm |
| intersection gap | 1.0 mm | 1.0 mm | 1.0 mm |
| acquisitions | 2 | 2 | 3 |

Post-contrast T1WI scans were acquired after intravenous administration of 0.1 mmol/kg of gadolinium-based contrast agents

Details of MRI parameters for Signa Excite HD 3.0 T; GE Medical Systems

|  | T1WI | T2WI | FLAIR |
| --- | --- | --- | --- |
| repetition time [TR] | 450 ms | 4,800 ms | 10,000 ms |
| echo time [TE] | 18 ms | 100 ms | 140 ms |
| flip angle [FA] | (-) | (-) | (-) |
| echo train length | (-) | 18 | (-) |
| inversion recovery time [TI] | (-) | (-) | 2400 ms |
| field of view [FOV] | 220 × 220 mm | 220 × 220 mm | 220 × 220 mm |
| matrix size | 288 × 192 | 512 × 320 | 288 × 160 |
| number of excitations [NEX] | 1 | 2 | 1 |
| section thickness | 6 mm | 6 mm | 6 mm |
| intersection gap | 1.0 mm | 1.0 mm | 1.0 mm |
| acquisitions | 2 | 1 | 2 |

Post-contrast T1WI scans were acquired after intravenous administration of 0.1 mmol/kg of gadolinium-based contrast agents
