# Supplementary material for: Super T2-FLAIR mismatch sign: a prognostic imaging biomarker for non-enhancing astrocytoma, IDH-mutant
Source: J Neurooncol. 2024 Jul 12;169(3):571–9. doi: 10.1007/s11060-024-04758-4 (PMC11341624; doi:10.1007/s11060-024-04758-4)
Supplement: Supplementary file 2 — Supplementary Material 2 [file 11060_2024_4758_MOESM2_ESM.docx]

| **Characteristics of the patients with astrocytoma, IDH-mutant according to the T2-FLAIR mismatch sign** | | | | | | | | | | |
| --- | --- | --- | --- | --- | --- | --- | --- | --- | --- | --- |
|  | **Our dataset (n=31)** | | | | | **TCGA (n=30)** | | | | |
|  | **T2-FLAIR mismatch sign** | | | | | | | | | |
|  | **Positive (n=17)** | | **Negative (n=14)** | | **P-value** | **Positive (n=9)** | | **Negative (n=21)** | | **P-value** |
| **Age at diagnosis (years)** | **40.8 ± 10.7** | | **40.4 ± 14.8** | | **0.9292** | **38.9 ± 14.6** | | **38.0± 12.1** | | **0.8636** |
| **Gender** |  |  |  |  | **0.2802** |  |  |  |  | **0.2360** |
| **Female** | **7** | **(41%)** | **3** | **(21%)** |  | **6** | **(67%)** | **8** | **(38%)** |  |
| **Male** | **10** | **(59%)** | **11** | **(79%)** |  | **3** | **(33%)** | **13** | **(62%)** |  |
| **KPS (%)** |  |  |  |  | **0.0671** |  |  |  |  |  |
| **< 90** | **1** | **(6%)** | **5** | **(36%)** |  |  |  |  |  |  |
| **> 90** | **16** | **(94%)** | **9** | **(64%)** |  |  |  |  |  |  |
| **WHO grade** |  |  |  |  | **0.0671** |  |  |  |  | **0.4311** |
| **2** | **16** | **(94%)** | **9** | **(64%)** |  | **7** | **(78%)** | **12** | **(57%)** |  |
| **3** | **1** | **(6%)** | **5** | **(36%)** |  | **2** | **(22%)** | **8** | **(38%)** |  |
| **Extent of resection** |  |  |  |  | **1.0000** |  |  |  |  |  |
| **Total** | **6** | **(35%)** | **4** | **(29%)** |  |  |  |  |  |  |
| **Non-total** | **11** | **(65%)** | **10** | **(71%)** |  |  |  |  |  |  |
| **Radiation therapy** | **11** | **(65%)** | **10** | **(71%)** | **1.0000** | **7** | **(78%)** | **17** | **(81%)** | **1.0000** |
| **Temozolomide** | **9** | **(53%)** | **10** | **(71%)** | **0.4607** |  |  |  |  |  |
| **FLAIR, fluid-attenuated inversion recovery; IDH, isocitrate dehydrogenase; KPS, Karnofsky Performance Status; TCGA, the cancer genome atlas; WHO, world health organization.**  *** p < 0.05** | | | | | | | | | | |

**Supplementary Material 2**
