# Supplementary figures and images for: Super T2-FLAIR mismatch sign: a prognostic imaging biomarker for non-enhancing astrocytoma, IDH-mutant
Source: J Neurooncol. 2024 Jul 12;169(3):571–9. doi: 10.1007/s11060-024-04758-4 (PMC11341624; doi:10.1007/s11060-024-04758-4)

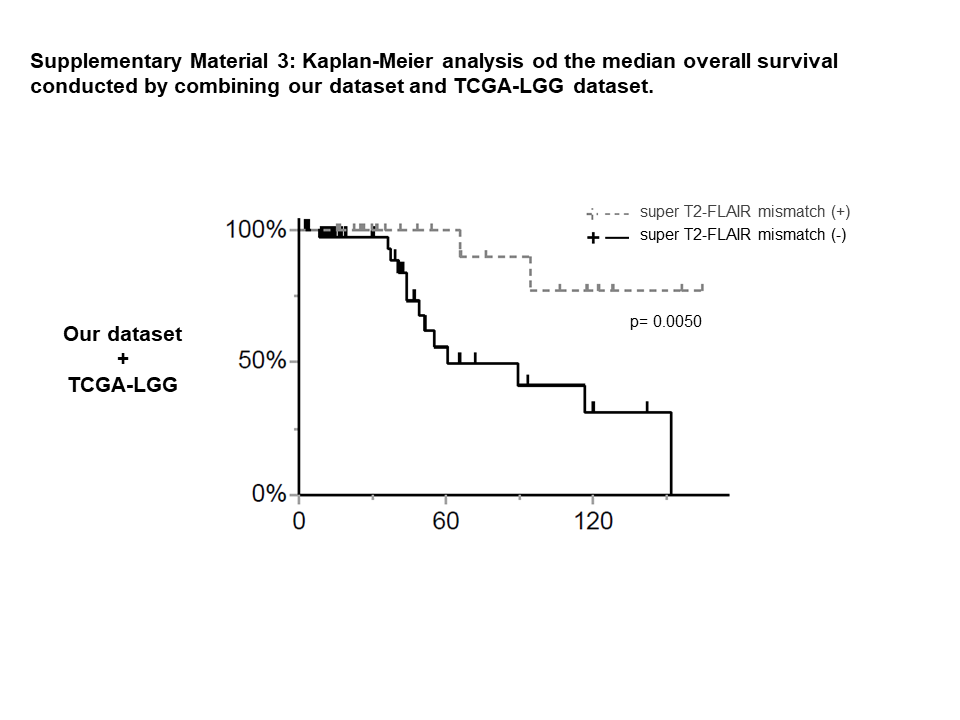

Supplement: Supplementary file 3 — Supplementary Material 3 [file 11060_2024_4758_MOESM3_ESM.tif]
